# Monitoring Antifungal Agents of *Artemisia annua* against *Fusarium oxysporum* and *Fusarium solani*, Associated with *Panax notoginseng* Root-Rot Disease

**DOI:** 10.3390/molecules24010213

**Published:** 2019-01-08

**Authors:** Yu-Nan Ma, Chuan-Jiao Chen, Qing-Qing Li, Fu-Rong Xu, Yong-Xian Cheng, Xian Dong

**Affiliations:** 1 College of Pharmaceutical Sciences, Yunnan University of Traditional Chinese Medicine, Kunming 650500, China; mayunan994727@163.com (Y.-N.M.); chenchuanjiao663@163.com (C.-J.C.); leecmm@163.com (Q.-Q.L.); xfrong99@163.com (F.-R.X.); 2 School of Pharmaceutical Sciences, Shenzhen University Health Science Center, Shenzhen 518060, China

**Keywords:** *Artemisia annua*, essential oil, *Panax notoginseng*, root rot, *Fusarium oxysporum*, *Fusarium solani*

## Abstract

Root rot of *Panax notoginseng* has received great attention due to its threat on the plantation and sustainable utilization of *P*. *notoginseng*. To suppress the root-rot disease, natural ingredients are of great importance because of their environment friendly properties. In this study, we found that the methanol extract from *Artemisia annua* leaves has strong antifungal effects on the growth of *Fusarium oxysporum* and *Fusarium solani* resulting into root-rot disease. Essential oil (EO) thereof was found to be the most active. GC-MS analysis revealed 58 ingredients and camphor, camphene, *β*-caryophyllene, and germacrene D were identified as the major ingredients. Further antifungal assays showed that the main compounds exhibit various degrees of inhibition against all the fungi tested. In addition, synergistic effects between *A*. *annua* EO and chemical fungicides were examined. Finally, in vivo experiments were conducted and disclosed that *P. notoginseng* root rot could be largely inhibited by the petroleum ether extract from *A*. *annua*, indicating that *A. annua* could be a good source for controlling *P. notoginseng* root-rot.

## 1. Introduction

*Panax notoginseng* is a perennial herb (Araliaceae). Its dry roots and rhizomes are used as medicinal parts known as San-Qi in Chinese. The main component of *P. notoginseng* is saponin. Relevant research showed that notoginseng saponin and heat-transformed ginsenoside have inhibitory activity against *Epidermophyton floccosum*, *Trichophyton rubrum*, and *Trichophyton mentagrophytes* [1]. Due to its great demands in cardiovascular diseases, it has been cultivated in Wenshan City of Yunnan Province for more than 400 years, where the planting area and yields have reached over 90% those of our country [2]. However, serious problems of continuous cropping obstacles (CROs) occur during cropping, resulting in decreased yields and loss of quality of *P*. *notoginseng* [3]. CROs could be resulted from multiple factors, of which, the root-rot disease caused by a single pathogen or a combination of pathogens could not be ignored. It is the most destructive and could result in yield reduction, no harvest, and a loss of active ingredients [4]. The pathogen fungi in the *P*. *notoginseng* rhizosphere have been investigated, indicating that *F*. *solani*, *F*. *oxysporum*, and *C. destructans* are the main pathogens of *P*. *notoginseng* root-rot disease [5,6,7]. Therefore, efforts targeting these fungal species might be helpful for discovering new agents against *P*. *notoginseng* root-rot disease. At the present, botanical fungicides have demonstrated their advantages owing to their eco-friendly property. In this regard, essential oils (EOs) of aromatic plants have recently received increased attention due to their low toxicity towards mammalians and high biodegradability [8]. China is rich in natural plant resources, which is one of the important ways to obtain new fungicides.

*Artemisia annua* L., a plant belonging to Asteraceae family, is a traditional Chinese herb mainly used for the treatment of malaria. Compounds such as artemisinin, artemisinic acid, and coumarins are characterized from this herb [9]. The medicinal ingredients in *A. annua* are non-volatile and volatile [10]. The non-volatile components are mainly artemisinin, artemisinic acid, coumarin, and other compounds, which are sesquiterpenoids compound. Some research revealed that Sesquiterpene lactones exhibit a wide range of biological activities, such as antitumor, anti-inflammatory, analgesic, antiulcer, antibacterial, antifungal, antiviral, antiparasitic, and insect deterrent [11]. Previous studies showed that *A*. *annua* EO has a broad antibacterial spectrum [12,13]. Moreover, the whole plant of *A*. *annua* has been used to prevent *P. notoginseng* root-rot by local people in Wenshan of Yunnan Province, China. The aim of the paper was to conduct an investigation to unveil the agents responsible for root-rot disease of *P. notoginseng*. In this paper, we describe our efforts on antifungal tests of *A*. *annua.*

## 2. Materials and Methods

### 2.1. Plants Cultivation

*P*. *notoginseng* seeds were seeded in sterile quartz sand and roseite with a 1:2 mixture in greenhouse. After germination, seedlings were cultivated under 20 °C with a relative humidity of 70% ± 10% and a photoperiod of 14 h day^−1^. The seedlings were supplied with full-strength Hoagland nutrient solution. The macronutrient composition of the Hoagland nutrient solution (in mg·L^−1^) was 40 N (as NH_4_NO_3_), 10 P (as KH_2_PO_4_), 40 K (as K_2_SO_4_ and KH_2_PO_4_), 57 Ca (as CaCl_2_), and 40 Mg (as MgSO_4_). The basal micronutrient composition (in mg·L^−1^) was 2.0 Fe (as Fe-EDTA), 0.2 B (as H_3_BO_3_), 0.5 Mn (as MnCl_2_·4H_2_O), 0.05 Mo [as (NH_4_)_6_Mo_7_O_24_·4H_2_O], 0.01 Zn (as ZnSO_4_·7H_2_O), and 0.01 Cu (as CuSO_4_·5H_2_O). Dicyandiamide, a nitrification inhibitor, was added to each nutrient solution to prevent oxidation of ammonium.

*A*. *annua* seeds were provided by the Yunnan Institute of Agricultural Science and Drug Research (Kunming, Yunnan, China). Seedlings were cultivated under 25 °C with full exposure. After the seedlings growth to 70 cm, the plants were harvested for use.

### 2.2. Preparation of Fungal Culture

The pathogenic fungi were isolated from the diseased *P*. *notoginseng* and identified as *Fusarium oxysporum* and *Fusarium solani*, by Sangon Biotech (Shanghai) Co., Ltd. (Shanghai, China). Then the two pathogens were cultured on potato dextrose agar (PDA) medium at 28 °C in the dark for seven days.

### 2.3. Pathogenicity Determination and Microscopic Observation of the Pathogenic Fungi

The conidia suspension of the fungi were prepared according to the method described by Hao [14]. The healthy *P*. *notoginseng* seedlings were submerged in a conidial suspension containing 1 × 10^6^ cfu/mL for 2 h. Then the negative control plants were treated with the sterile water. After infection, the *P*. *notoginseng* plants were continually cultivated in the greenhouse. After approximately 20 days of growth, the numbers of *P*. *notoginseng* diseased plants were counted. The mycelium and conidia of the two pathogens were put into a temporary slide for microscopic observation. Then the morphology of hyphae and conidia were observed under optical microscope (Olympus Corporation, Tokyo, Japan).

### 2.4. Preparation of Methanol Crude Extract from A. annua

The roots, stems and leaves of *A*. *annua* were completely separated and dried under natural conditions, then the samples were crushed into powders with a pulverized machine. The different parts powders (50 g) were put into a triangle bottle, and 450 mL different concentrations of methanol (0%, 25%, 50%, 75%, 100%) were added. A 600 W, 40 kHz sonicator (Model: 15D793, Ningbo Scientz Biotechnol Co., Ltd., Ningbo, Zhejiang, China) was used for ultrasound-assisted extraction. The samples were ultrasonic for 2 h and static for half an hour at 37 °C. During the extraction process, the sample container was held in a thermostat-controlled water bath. Then the extraction processes were repeated three times. After extraction, the crude extract was filtered and stored at 4 °C for use.

### 2.5. Determination of Antifungal Activity of A. annua Crude Extract In Vitro

The oxford cup method was adopted to determine the effects of *A. annua* crude extract on the growth of pathogens mycelia [15]. Two pathogens were cultured on PDA medium for five days, then a 5 mm-wide disc of fungi containing agar was excised from the culture margins, which was placed in the middle of the petri dishes. The four oxford cups were placed in the same distance around the fungi discs, which the distance between the oxford cup and the middle of petri dish was 25 mm. Then the crude extract was filtered through 0.22 µm hydrophobic membranes, and the sterile filtrate extract was obtained. The filtrate extract of 200 µL was added to every oxford cup. Different concentrations of methanol were used as the negative control. Each treatment was repeated three times. Then the pathogens were cultured in the incubator at 28 °C for seven days, and the diameter of colony was measured based on the average value of two perpendicular diameters. The inhibitory effect of the crude extract on pathogenic fungi was shown by histogram.

### 2.6. Preparation of EO and Non-Volatile Fractions from A. annua Leaves

The sample of 100 g *A. annua* leaves powder was placed in a flask with a round bottom, and 1200 mL distilled water was added. Then the sample was soaked for half an hour. EO was extracted by steam distillation and distilled at 100 °C for 6 h. The steam distillation apparatus consisted mainly three parts, which were steam equipment, condensation equipment and oil collection equipment. After the extraction, the EO was collected, dehydrated with anhydrous sodium sulfate and stored in a brown sample bottle, named as II (EO). After extraction, the residues were filtered and the water part was concentrated, which was named as III (water soluble part). The filtered residues were extracted with methanol by ultrasonic twice, and the mixtures were concentrated and dried, named as IV. An aliquot of sample IV was suspended in water and extracted with equal volume of chloroform thrice, and the chloroform layer was concentrated and dried to obtain V. The water layer was concentrated to be VI. Sample was obtained by direct ultrasonic extraction using methanol, which was VII. All the samples were stored at −20 °C for further analysis.

### 2.7. Antifungal Effect of Fractions II-VII from A. annua Leaves

Fractions II-VII obtained from *A*. *annua* leaves were dissolved in a mixture solution of 10/1000 DMSO and 1/1000 Tween 80, and the final concentration of components was 50 mg/mL. Meanwhile, 10/1000 of DMSO and 1/1000 of Tween 80 mixed solution (I) was used as the negative control. The effects of *A*. *annua* leaves different extract on pathogenic fungi were carried out according to the oxford cup method above.

### 2.8. Analysis of A. annua EO by GC-MS

The EO were analysis by GC-MS with an HP-5 MS quartz capillary column (30 mm × 0.25 mm × 0.25 mm) (Agilent Technologies, Santa Clara, CA, USA) [16]. Helium (1.2 mL/min) was used as carrier gas. The injector temperature was set at 250 °C. An initial column oven temperature of 80 °C was elevated to 240 °C at a rate of 3 °C/min and held for 50 min. The mass spectrometer (MS) conditions were as follows: transfer line temperature at 250 °C, ion source at 230 °C, acquisition mass range 30–500, and the ionization potential at 70 eV. Then the relative peak area percentages of the different compounds were calculated based on the FID data. The identification of the analyzed compounds were accomplished by comparing their mass spectra with those of authentic compounds available from computerized spectral database (NIST).

### 2.9. Determination of Minimum Inhibitory Concentration (MIC)

The MIC values test was carried out by 96-well plates [17]. The *A. annua* EO, main components and two kinds of chemical fungicides (Flutriafol and Hymexazol) were diluted with 20/1000 of DMSO and 1/1000 of Tween 80 solutions by double dilution method. *A. annua* EO had 12 groups of solution with a final concentration range (37.50–0.04 mg/mL), then the chemical fungicides and main compounds (l-camphor; dl-camphor, *β*-caryophyllene and camphene) with a final concentration range (2.5–0.005 mg/mL) were also prepared. Conidia suspension was prepared with a 1/4 PDB culture. The number of microconidia was measured by blood cell counting board, with final concentration of 1 × 10^4^ cfu/mL. A mixture formed from filter-sterilized EO (50 μL) and suspension of the fungi (150 μL) were added to the wells of 96-cell plate. Then 150 μL suspension and 50 μL solution that included 20/1000 DMSO and 1/1000 of Tween 80 was added to the wells as a negative control. The 96-well plates were kept at a constant temperature of 28 °C for 36 h. After the incubation period, the wells were checked for fungi growth. The absorbance of each well was measured at 595 nm by using a microplate reader (Model: 1510, Thermo Fisher Scientific, Waltham, MA, USA). 

### 2.10. Synergistic Effects Observation of A. annua EO and Chemical Fungicides

The checkerboard microdilution assay was used to evaluate the mixed solution effect [18]. MIC values were measured according to the above step. Then a two dimensional checkerboard with serial two fold dilutions of each compound was used, and the concentration ranged from 1/16 × MIC to 8 × MIC. Different concentrations of EO and different concentrations of hymexazol or flutriafol (each 25 μL) were combined into the each well. Each well contained a mixture of two drugs with different concentrations, and suspension of the fungi (150 μL) was added to the well. The 150 μL suspension of the fungi and 50 μL solution that included 20/1000 DMSO and 1/1000 of Tween 80 as a negative control. Then the 96-well plates were kept at a constant temperature of 28 °C for 36 h. After the incubation period, the wells were checked for fungi growth. The absorbance of each well was measured at 595 nm by using microplate reader (Model: 1510, Thermo Fisher Scientific, Waltham, MA, USA). MIC determination of combined drugs use: the concentration of drugs corresponding to wells growing without fungi was used as the MIC of EO combined with hymexazol or flutriafol.

FIC of *A. annua* EO = MIC of *A. annua* EO in combination with chemical fungicides/MIC of *A. annua* EO alone, FIC of chemical fungicides = MIC of antifungal in combination with EO/MIC of chemical fungicides. FICI = FIC of *A. annua* EO + FIC of chemical fungicides. The types of effects were classified as follow: FICI ≤ 0.5, synergism effect; 0.5 < FICI ≤ 1, additive effect; 1 < FICI ≤ 4, irrelevant effect and FICI > 4, antagonism [19].

### 2.11. Preparation of Petroleum Ether Extract from A. annua Leaves

The *A*. *annua* leaves powder was extracted by 85% ethanol with reflux, then ethanol was distilled to obtain concentrated solution. The equal volume of water and petroleum ether were added to the concentrated solution soaking for 12 h, then the petroleum ether layer was extracted. The operation was repeated for three times. Then the crude petroleum ether extract from *A*. *annua* was prepared by decompression recovery of petroleum ether. 

### 2.12. The In Vivo Effect of Petroleum Ether Extract from A. annua on the Incidence of P. notoginseng

The sterilized quartz sand and roseite were mixed in proportion of 2:1 as *P*. *notoginseng* culture medium. The petroleum ether extract was mixed into the matrix, and the concentrations were 0.25 mg/g and 0.5 mg/g. The healthy *P*. *notoginseng* seedlings were submerged in a mixed conidial suspension containing 1 × 10^6^ cfu /mL of both *F*. *oxysporum* and *F*. *solani* for 2 h. Six *P. notoginseng* plants were planted into a pot. The culture medium without petroleum ether extract was set as a negative control. Three replicates was performed. After 20 days of infection by the pathogenic fungi, the plants were graded for severity of wilt disease as 0 (not showing chlorosis), 1 (the stem soft), 2 (the stem fallen, but the leaf not wilted), and 3 (plant wilting). The wilting rate (%) = the number of wilting plants/total number of plants × 100. Disease index = ∑(rating × number of plants rated)/(total number of plants × highest rating) × 100 [20].

### 2.13. Statistical Analysis

Statistical analysis was performed with SPSS Statistics 19.00 (SAS Institute, Cary, NC, USA) by using one way ANOVA and Duncan’s multiple comparisons test.

## 3. Results

### 3.1. Pathogenic Determination and Microscopic Observation of Pathogenic Fungi

**Figure 1:**
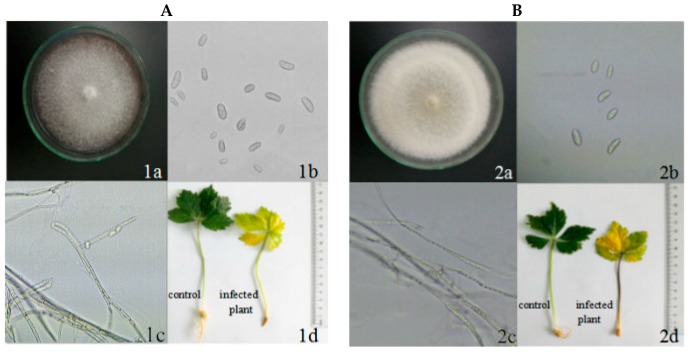
Microscopic observation of *F*. *oxysporum, F*. *solani* and infection of *P*. *notoginseng*. (**A**): *F*. *oxysporum*. (**B**): *F*. *solain.* (a) colonial morphology; (b) microconidia; (c) mycelium form; (d) *P*. *notoginseng* plants infected by pathogenic fungi.

**Figure 2:**
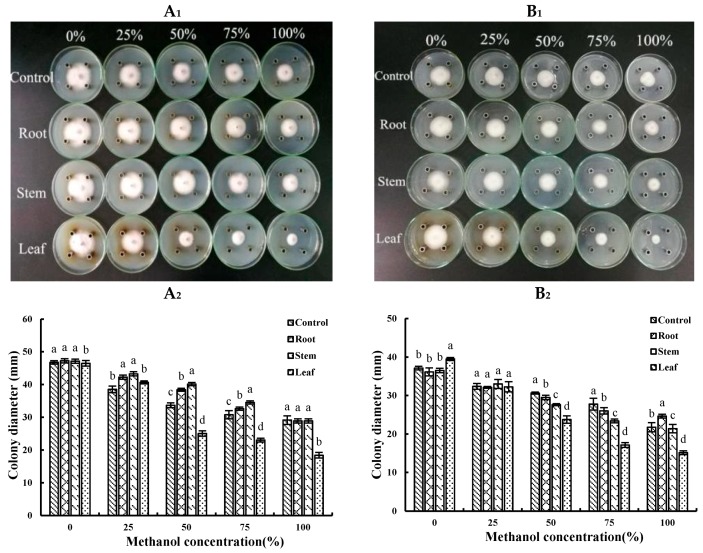
(**A_1_**): Effects of different concentrations of methanol crude extracts from *A*. *annua* against *F*. *oxysporum*, and the mycelium growth for 5 days. (**B_1_**): Effects of different concentrations of methanol extracts from *A*. *annua* on *F*. *solani*. (**A_2_**): The colony diameter (mm) of *F*. *oxysporum* by treated with different crude extracts. (**B_2_**): The colony diameter (mm) of *F*. *solani* by treated with different crude extracts. Note: Different letters represent significant differences (*p* < 0.05) among different plant parts at the same methanol concentration, while with the same letters mean no significant difference (*p* > 0.05).

Two pathogenic fungi were cultured on PDA medium for seven days. *F. oxysporum* grew into a round colony (Figure 1, 1a) with an average diameter of 70 mm, and the colonies changed from white to pink. The hyphae were villous. Under microscope, small conidia and hyphae could be clearly seen. The small conidia was oval or round (Figure 1, 1b), and the size was (7.3−18 µm) × (3.3−5.7 µm). The mycelium was abundant and the surface was smooth, and small conidia were produced (Figure 1, 1c). The colony of *F. solani* was round, and the mycelium was white or pale yellow. The mycelium of *F. solani* was flocculent and grew vigorously (Figure 1, 2a). Small conidia was sickle shaped or long column shaped (Figure 1, 2b), with a size of (10−18 µm) × (3−5 µm). The mycelium was straight, long, and smooth (Figure 1, 2c). After *P. notoginseng* pathogen infection, the above ground part of the plants was short and the leaves turned yellow. The surface of stem was dry, and the root rot was obvious (Figure 1, 1d and 2d).

### 3.2. Inhibitory Effect of Methanol Crude Extract from A. annua on Pathogenic Fungi

Inspired by the wisdom of local people and to tracking antifungal agents of *A. annua,* first, the methanol extracts of different plant parts were prepared and examined for their inhibition on the growth of the fungi. As shown in Figure 2A_1_,A_2_). The extracts of the roots and the stems from *A*. *annua* have no inhibitory effect on *F*. *oxysporum*, whereas the inhibition rate of the leaves extract (with 50% methanol) on *F*. *oxysporum* is 25.37% and is up to 36.94% with the pure methanol extract. With the increase of concentration of methanol extract, the antifungal potency of *A. annua* leaves extract also increases. Similar tendency is observed for *F*. *solani* (Figure 2B_1_,B_2_). In contrast, the extracts from the other parts rather than leaves are almost inactive against *F*. *solani.* And the methanol extract from the leaves is found to be the most active toward *F*. *solani*, revealing that antifunfal agents are mainly present in *A. annua* leaves.

### 3.3. Inhibitory Effect of Fractions II-VII from A. annua Leaves on the Fungi

**Figure 3:**
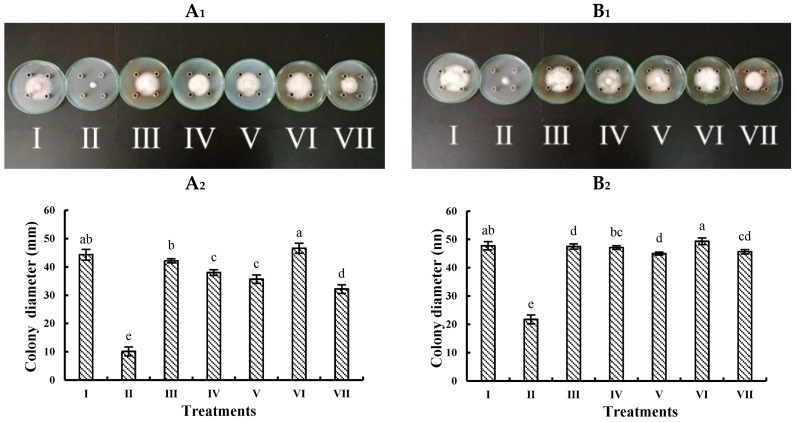
Inhibitory effect of fractions II−VII from *A*. *annua* on *F*. *oxysporum* and *F*. *solani*. **A_1_**: The mycelium growth of *F*. *oxysporum* for 5 days. **B_1_**: The mycelium growth of *F*. *solani* for five days. **A_2_**: The colony diameter (mm) of *F*. *oxysporum* under different components. **B_2_**: The colony diameter (mm) of *F. solani* under different components. I: Negative control; II: EO; III: Water soluble extract; IV: Methanol soluble extract; V: Chloroform soluble extract; VI: Water soluble extract; VII: Methanol soluble extract directly extracted by ultrosonic method. Note: In the same figure, different letters represent significant differences (*p* < 0.05) among different treatments, while with the same letters mean no significant difference (*p* > 0.05).

To unveil the active fractions, EO and non-volatile fractions were rationally prepared and examined. As shown in Figure 3, the EO (II) from *A*. *annua* leaves has the most significant inhibitory effect on the mycelial growth of *F*. *oxysporum* (Figure 3A_1_,A_2_) and *F*. *solani* (Figure 3B_1_,B_2_) with the inhibition rates of 77.16% and 54.64%, respectively. Secondary is fraction VII extracted directly by ultrasound. Fractions IV and V have relatively weak suppression on the pathogenic fungi, whereas the inhibitory effects of water soluble (III) and chloroform soluble (VI) on two pathogens are almost negligible. Collectively, the pathogens are sensitive to *A. annua* EO, disclosing that antifungal activity of *A. annua* leaves is mainly resulted from volatile components rather than non-volatile substances.

### 3.4. Analysis of A. annua EO Chemical Components by GC-MS

To get an insight into the chemical profile of *A. annua* volatile mixtures, the *A. annua* EO was obtained by steam distillation described above with a yield of 0.18%, which was subsequently submitted to GC-MS analysis. As shown in Table 1, a total of 58 compounds are identified, accounting for 89.39% of the whole EO composition. The principal compound in *A. annua* EO is camphor (23.48%), followed by germacrene D (9.06%), *β*-caryophyllene (5.39%), and camphene (4.42%), respectively. 

### 3.5. MIC Determination of A. annua EO and Main Compounds

To understand active compounds responsible for antigungal activity of EO, the MIC of *A. annua* EO and the abundant compounds were compared. As shown in Table 2, l-camphor; dl-camphor, *β*-caryophyllene, and camphene are all active toward *F*. *oxysporum* with MIC values of 0.11 mg/mL, 0.14 mg/mL, 0.13 mg/mL, and 0.16 mg/mL, respectively, comparable to those of *A. annua* EO and two positive controls (flutriafol and hymexazol). For *F*. *solani*, it is also sensitive to l-camphor; dl-camphor, *β*-caryophyllene, and camphene with respective MIC value of 0.31 mg/mL, 0.16 mg/mL, 0.23 mg/mL, and 0.22 mg/mL, equivalent to those of EO (0.37 mg/mL), flutriafol (0.11 mg/mL), and hymexazol (0.16 mg/mL).

### 3.6. Evaluation of the Synergistic Action of A. annua EO and Chemical Fungicides

The FIC indices in combination with *A. annua* EO and hymexazol or flutriafol were given in Table 3. According to the FICI values, the combined use of *A. annua* EO and flutriafol shows additive (0.75) inhibitory effect on *F. solani*, while the effects on *F. oxysporum* is irrelevant (1.25). At the same time, the results reveal the additive effect (0.63) on inhibiting *F. oxysporum* and synergistic (0.38) effect on inhibiting *F. solani*, when *A. annua* EO combined with hymexazol.

### 3.7. Effect of the Petroleum Ether Extract from A. annua on the Incidence of Infected P. notoginseng

In vivo tests were needed to check whether the in vitro active agents make sense for the living *P. notoginseng*. To satisfy the requirement of in vivo experiment, the petroleum ether extract from *A. annua* leaves, which simulates the components of *A. annua* EO, was prepared and mixed into the culture medium. As shown in Table 4, the petroleum ether extract could reduce the occurrence of *P. notoginseng* root-rot in a certain concentration. When the concentration of the petroleum ether extract is 0.5 mg/g, the incidence of the plants is 25.00%, which can decrease by 47.22%. Additionally, the disease index of the infected plants is 13.10, which is also significantly lower than that of the negative control.

## 4. Discussion

Root-rot is a world-wide soil-borne disease, which can seriously damage many crops and medicinal plants and limit the continuous development of agriculture. *P. notoginseng* root-rot can occur in one- or two-year growth of *P. notoginseng*, whereas the occurrence rate of *P. notoginseng* older than two years is more severe [21]. Chemical fungicides are a double-edged sword which guaranteed the agricultural development but also bring serious pollution and harm to ecology and environment or human health [22]. Flutriafol and hymexazol are economically important agricultural chemicals, which are able to control several diseases affecting a wide range of crops. However, their high mobility potential in the soil makes them problematic fungicides. Indeed, flutriafol is a potentially toxic chemical fungicide, which may disrupt fertility in women and affect the endocrine system [23]. In contrast to chemical fungicides, botanical fungicides are wanted in agriculture. In consequence, it is very urgent to look for new compounds from natural material to develop new kinds of high efficiency, low toxicity fungicides. In the study, the results showed that *F*. *oxysporum* and *F*. *solani* have strong pathogenicity to *P. notoginseng*. After infection for a period of time, *P*. *notoginseng* appears the disease symptoms with yellowing of leaves and rot of roots (Figure 1). By comparing the potency of the methanol extracts with roots, stems, and leaves from *A*. *annua*, it was found that the extracts from *A. annua* with different methanol concentrations have various antifungal activities. The antifungal effect of *A*. *annua* leaves extracted by pure methanol is the strongest (Figure 2). When the methanol concentration reached 100%, the antifungal activity on various fungi is the most significant. At the same time, when the extraction method is the same, the antifungal activity of leaves extract is the best, while the effect of roots is the worst. Some studies showed that the ethanol extract from *A. annua* could inhibit mycelium growth of *Cytospora chrysosperma* and *Guignardia laricina*, and its inhibition rates are 100% and 91.74%, respectively [24]. Therefore, an effort was made to know what kinds of substances in *A*. *annua* leaves are active. Then solvents with different polarity were used for fractionation and antifungal evaluation. The results reveal that *A. annua* EO is the most active, whereas the inhibitory effects of water soluble substances are negligible (Figure 3). By comparing the potency of different fractions, it might infer that EO is responsible for the major antifungal activity of *A*. *annua*. According to the others’ studies, the antifungal activity of *A. annua* EO was evaluated against economically important foliar and soil borne fungal pathogens of tomato, which has revealed that *A. annua* EO is active against *Sclerotinia sclerotiorum, Botrytis cinerea*, *Phytophthorainfestans*, and *Verticillim dahliae* [25]. They also have suggested that *A. annua* EO could be used as potential antifungal agents to treat or prevent the pathogenic fungi.

In the previous study, EOs from either *Alpinia officinarum* Hance and *Amomum tsao-ko* (*Zingiberaceae*) are found to have a good inhibition against the *F. oxysporum*, *F. solani*, and *C. destrutans* [26], and the EOs of *Alpinia Katsumadai* Hayata and *Zingiber officinal* Roscoe also have significant reductions in the mycelium growth of the pathogen in vitro [27]. This is corroborated by our findings. The results uncover that the main ingredients are camphor, camphene, germacrene D, and *β*-caryophyllene (Table 1), which accounted for 42.35% of the total EO. Perazzo et al. has extracted EO from *A. annua* and identified 25 compounds including camphor (43.5%), macrocarbene D (16.0%), *trans*-pinocarveol (11.0%), and herb-cymene (9.0%) [28]. The EO in the analysis revealed a great variability both in the qualitative and quantitative composition. Chemical profile is generally influenced by the harvesting season, fertilizer, and the pH of soils, the choice and stage of drying conditions, the geographic location, chemotype or subspecies, and choice of part plant or genotype or extraction method [29]. Some study showed that the yield and main components of the EOs extracted from *A. annua* are varied at different flowering stages. The highest amounts of the main components were recorded in the full flowering stage, which main compounds are artemisia ketone (28.30–37.15%), camphor (18.00–23.30%), and 1,8-cineole (9.00–10.39%) [30]. Then, the MIC values against *F*. *oxysporum* and *F*. *solani* were determined with the *A*. *annua* EO and the main compounds (Table 2). The results prove that the pathogenic fungi show a high sensibility to *A*. *annua* EO and the main compounds, with MIC ranging from 0.09 to 0.37 mg/mL.

Previous study showed that 14-hydroxyltajixanthone, which was isolated from the solidsubstrate fermentation culture of *Emericella* sp. XL029 associated with the leaves of *P. notoginseng*, exhibited significant activity against *Rhizoctonia cerealis*, *Fusarium oxysporum*, and *Physalospora piricola*, with MIC value of 25 μg/mL [31]. Even though the MIC results varied among the tested strains, in most cases, the MIC values of *A*. *annua* EO and the main compounds were equivalent to the MIC values of chemical fungicides. The *A*. *annua* EO is a complex mixture of different compounds, so it is difficult to attribute the EO antifungal activity to a single or a particular constituent. In addition to the major components, also minor components may make a significant contribution to the antifungal activity. Following the results above, in order to further understand the antifungal mechanism of EO from *A. annua*, the extract can be isolated and purified, and the antifungal mechanism with the structure-activity relationship of the active substance could be discussed in more depth in the future. Research showed that the interactions between antifungal substances could be divided into four types: synergistic, additive, irrelevant, and antagonistic [32]. In our experiment, for the purpose of improving the efficiency of fungi inhibition, the antifungal activity between *A. annua* EO and the chemical fungicides of hymexazol and flutriafol were studied. The results indicates that when *A. annua* EO was used in combination with hymexazol or hymexazol, the growth of tested strains could be effectively inhibited at a much lower concentration (Table 3), and the synergistic or additive effects were exhibited while the antifungal effect was not affected. The existence of synergies could greatly reduce the use of chemical pesticides, which can not only reduce the cost, ensure the safety, but also reduce the fungicide residue in traditional Chinese medicine. Since the difference in quantity and quality between the compound composition of EO and the active compound group of single drug, a new active compound group is formed, which can lead to some new different pharmacodynamics [33]. Therefore, the pharmacological effects of the new ingredients need to be further studied. For further simulating the antifungal effect of *A. annua* EO in vivo, the petroleum ether extract was prepared from *A. annua* leaves to conduct an in vivo experiment. The results revealed that the petroleum ether extract could reduce the incidence of infected *P*. *notoginseng* plants in a certain concentration range (Table 4). The major inhibitory effect is resulted from the EO components of the petroleum ether extract (Figure S1). The results of in vitro experiments show that the *A*. *annua* EO has certain inhibitory effect on the growth of mycelia and the germination of conidia. Therefore, it is speculated that the addition of *A*. *annua* EO to the soil may inhibit the pathogenic fungi germination of pathogenic and mycelial growth, which could inhibit the pathogenic fungi from infecting the plants of *P*. *notoginseng*. Finally, it would reduce the plants incidence and disease index. Nevertheless, the EO effects on the other soil microbial and plants need to be further studied. Some studies have indicated that botanical fungicides, such as matrine and garlicin, are likely to be a panacea for sustainable agricultural development and sustainable stability of the ecological environment survival despite their mild effects [34]. *A. annua* is widely distributed in China and has a high yield. Particularly, its EO has a good inhibitory effect on bacteria and fungi. The present findings of antifungal property of EO from *A*. *annua* against root rot pathogenic fungi might lend supports for developing *A*. *annua* as a natural fungicide especially beneficial for *P*. *notoginseng* root rot disease.

## 5. Conclusions

Our previous field experiments found that covering *A*. *annua* on the surface of soils could promote the growth of *P. notoginseng*, increase the content of chlorophyll, and promote plant height and dry weight of roots. The incidence of the disease was significantly lower than that of *P*. *notoginseng* without covering *A*. *annua,* which greatly inhibited the occurrence of yellow rot [35]. This observation inspired us to clarify why *A*. *annua* could reduce the root rot disease of *P. notoginseng*. In this study, it was found that *F*. *oxysporum* and *F*. *solani* have strong pathogenicity to *P. notoginseng*. The extracts from *A*. *annua* leaves other than the roots, stems are the most active. Moreover, volatile components from *A*. *annua* EO were found to be the strongest. GC-MS analysis reveal the chemical profile of *A. annua* EO, and further antifungal tests disclose antifungal potency of camphor, camphene and *β*-caryophyllene against *F*. *oxysporum* and *F*. *solani*, equivalent to the MIC values of chemical fungicides. This study also found that *A. annua* EO could reduce the concentration of hymexazol or hymexazol, two chemical fungicides, indicating their synergistic effects. Finally, an in vivo experiment revealed that the petroleum ether extract (simulating EO) could reduce the incidence of infected *P*. *notoginseng* plants, in accordance with our previous field experiments. Our efforts resulted in EO of *A*. *annua* and the main compounds thereof are active agents towards fungal species associated with *P. notoginseng* root-rot disease, which will be beneficial for the pharmaceutical industry of *P. notoginseng*.

## Figures and Tables

**Table 1:** Chemical composition and content of EO from *A. annua.*

NO	R T (min)	Compounds	Percent Area (%)
1	2.62	2-Hexenal	0.25
2	3.57	*α*-Pinene	1.67
3	3.79	Camphene	4.42
4	4.22	*β*-Pinene	1.28
5	4.33	Myrcene	0.40
6	5.04	*p*-Cymene	0.54
7	5.21	Cineole	1.84
8	5.76	Artemisia ketone	1.05
9	5.99	*trans*-Sabinene hydrate	0.13
10	6.49	*α*-Terpinolene	0.89
11	7.53	*α*-Campholene aldehyde	0.13
12	8.26	Camphor	23.48
13	8.65	Pinocarvone	0.42
14	8.75	Borneol	0.67
15	9.09	D-Carvone	0.53
16	9.49	*α*-Terpineol	0.19
17	9.69	Myrtenol	0.54
18	10.42	(-)-Carveol	0.23
19	14.65	*trans*-Chrysanthenylacetat	0.32
20	15.44	Eugenol	0.27
21	15.61	cis-Carvyl acetate	0.15
22	16.15	*α*-Copaene	0.96
23	16.54	3-Methyl-butanoic acid phenylmethyl ester	0.69
24	17.01	*cis*-Jasmone	0.19
25	17.88	Caryophyllene	5.39
26	18.17	*β*-Gurjunene	0.13
27	19.09	*α*-Caryophyllene	0.45
28	19.23	*trans*-*β*-Farnesene	1.64
29	19.37	*γ*-Curcumene	0.27
30	19.96	Selina-4(15),7(11)-diene	1.39
31	20.26	Germacrene D	9.06
32	20.42	*β*-Selinene	3.19
33	20.76	Bicyclogermacrene	1.43
34	21.42	*γ*-Cadinene	0.50
35	21.76	*δ*-Cadinene	0.69
36	23.28	Nerolidol	0.51
37	23.67	Nootkatone	1.14
38	23.98	5-Butyl-3-methyl-1,2,3,8A-tetrahydroindolizine	1.25
39	25.15	*α*-Cedrol	0.51
40	25.73	6,7-Dimethyltetralin	0.84
41	25.89	Cadina-3,9-diene	1.69
42	26.18	*tau*-Muurolol	1.10
43	26.65	(-)*-a*-Cadinol	1.36
44	27.01	Isopyrethrone	1.02
45	27.84	Ledane	2.14
46	28.25	Caryophylla-2(12),6(13)-dien-5α-ol	0.42
47	28.34	7-Hydroxy-6-propanoylcoumarin	0.52
48	28.98	Zierone	0.99
49	30.01	10,10-Dimethyl-4-acetyl-tricyclo[5.2.2.0(1,5)]Decane	0.59
50	30.32	(±)-Nootkatone	0.86
51	30.54	6*β*-Hydroxy-1,10-dehydrofuranoeremophil-9-one	0.61
52	30.81	Cyclogeranyl bromide	3.63
53	31.05	4-Phenyl-2-thiazolylamine	0.83
54	32.48	1,5-Dimethyl-2,6,7,7a-tetrahydro-1H-indene-3-carbaldehyde	1.01
55	33.24	Artemisinic acid	0.11
56	35.79	3-Methyl-7-methoxy-2-benzopyran-1(1H)-one	1.73
57	37.16	Hexadecanoic acid	0.65
58	37.99	Dispiro[5,2,5,2]hexadecan-1-one	0.49

Note, RT: Retention time.

**Table 2:** MIC values of *A. annua* EO and compounds against fungi (mg/mL).

	EO	Flutriafol	Hymexazol	l-Camphor	dl-Camphor	*β*-Caryophyllene	Camphene
*F. oxysporum*	0.22 ± 0.03 ^a^	0.10 ± 0.02 ^b^	0.12 ± 0.02 ^b^	0.11 ± 0.02 ^b^	0.14 ± 0.04^b^	0.13 ± 0.01 ^b^	0.16 ± 0.03 ^a b^
*F. solani*	0.37 ± 0.08 ^a^	0.11 ± 0.02 ^c^	0.16 ± 0.00b ^c^	0.31 ± 0.06 ^a b^	0.16 ± 0.05 ^b c^	0.23 ± 0.03 ^a b c^	0.22 ± 0.03 ^a b c^

Note: Each data point represents the mean ± SD of three replicates. Different letters represent significant differences. (*p* < 0.05) among different treatments on the same line, while with the same letters mean no significant difference (*p* > 0.05).

**Table 3:** The FICI values by combining *A. annua* EO with chemical fungicides.

	EO + Flutriafol		EO + Hymexazol	
	FICI	Results	FICI	Results
*F. oxysporum*	1.25	Irrelevant	0.63	Additive
*F. solani*	0.75	Additive	0.38	Synergic

FICI ≤ 0.5, synergism effect; 0.5 < FICI ≤ 1, additive effect; 1 < FICI ≤ 4, irrelevant. effect; FICI > 4, antagonism.

**Table 4:** Effect of the petroleum ether extract from *A*. *annua* on disease index and incidence of *P*. *notoginseng* plants infected by pathogenic fungi.

Difference Targets	Concentration (mg/mL)		
	Control	0.25	0.5
Disease index	72.23 ± 9.58 ^a^	16.67 ± 9.60 ^b^	13.10 ± 7.42 ^b^
Incidence (%)	72.22 ^a^	27.78 ^b^	25.00 ^b^

Note: In the same line, different letters mean significant difference (*p* < 0.05), while with the same letters mean no significant difference (*p* > 0.05).

## Supplementary Materials

The following are available online at http://www.mdpi.com/1420-3049/24/1/213/s1.
