# Supplementary material for: Monitoring Antifungal Agents of Artemisia annua against Fusarium oxysporum and Fusarium solani, Associated with Panax notoginseng Root-Rot Disease
Source: Molecules. 2019 Jan 8;24(1):213. doi: 10.3390/molecules24010213 (PMC6337599; doi:10.3390/molecules24010213)
Supplement: Supplementary file 1 [file molecules-24-00213-s001.pdf]

Supporting Information

Material and Methods

The EO was extracted from the petroleum ether extract (2.6 g) of *A. annua.*Then 0.95 g of the EO was extracted by steam distillation with 100 mL distilled water. The non-volatile component was 1.43 g. The EO and non-volatile components obtained from *A. annua* were respectively dissolved in a mixture solution of 10/1000 DMSO and 1/1000 Tween 80, and the final concentration was 50 mg/mL. Then the effect of different parts on pathogens were carried out according to the oxford cup method above.

Results and Analysis

The yield of EO was obtained by hydrodistillation of an air-dried sample with a yield of 36.54% (w/w) from the petroleum ether extract. From the experimental results (Figure 5), it could be concluded that the colony diameters from non-volatile components on the two pathogenic fungi are much small compared with the control group. After treatment with non-volatile components, the colony diameters for *F. oxysporum* and *F. solani* are 49.00 mm and 46.17 mm, respectively (Figure 5 A_2, B_2). However, with the treatment by EO, the diameter of the colonies for *F. oxysporum* and *F. solani*was are 28.83 mm and 29.33 mm. The colony diameter with the treatment of EO is greatly reduced, compared with the non-volatile components (Figure 5 A_1, B_1). Therefore, it is further proved that EO played an important role in antifungal activity, and the inhibitory effect of the EO on the pathogenic fungi is remarkable. This comparison provides the basis for *in vivo* concentration design of the petroleum ether extract.

A_1

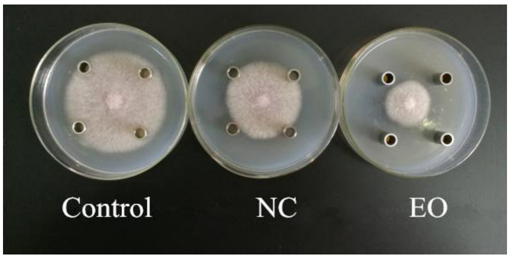

B_1

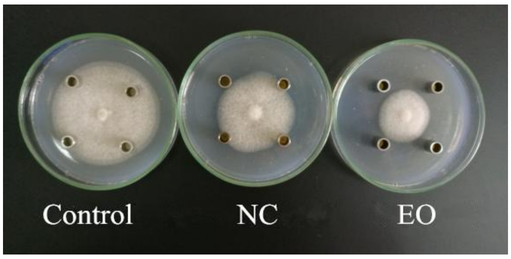

A_2

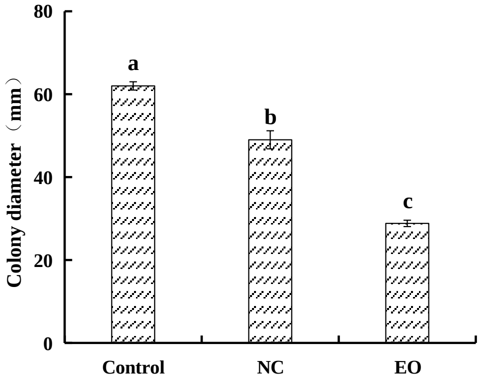

B_2

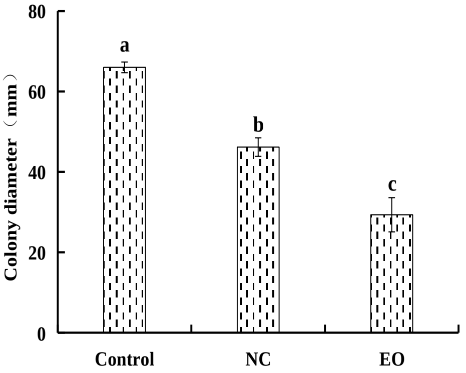

Figure S1. Inhibitory effect of non-volatile components and EO from *A. annua* on *F. oxysporum and F. solani*. Note, NC: Non-volatile components, EO: Essential oil. A_1: The mycelium growth of *F. oxysporum* for 5 days. B_1: The mycelium growth of *F. solani* for 5 days. A_2: The colony diameter of *F. oxysporum* under different components. B_2: The colony diameter of *F. solani* under different components.
